# Small Molecule Inhibitors Specifically Targeting the Type III Secretion System of *Xanthomonas oryzae* on Rice

**DOI:** 10.3390/ijms20040971

**Published:** 2019-02-23

**Authors:** Hui Tao, Su-Su Fan, Shan Jiang, Xuwen Xiang, Xiaojing Yan, Lian-Hui Zhang, Zi-Ning Cui

**Affiliations:** 1 State Key Laboratory for Conservation and Utilization of Subtropical Agro-bioresources, Integrative Microbiology Research Centre, Guangdong Province Key Laboratory of Microbial Signals and Disease Control, South China Agricultural University, Guangzhou 510642, China; htao2018@126.com (H.T.); scaujs@stu.scau.edu.cn (S.J.); xxw@stu.scau.edu.cn (X.X.); lhzhang01@scau.edu.cn (L.-H.Z.); 2 State Key Laboratory for Biology of Plant Diseases and Insect Pests, Institute of Plant Protection, Chinese Academy of Agricultural Sciences, Beijing 100193, China; 1986fansusu@163.com (S.-S.F.); yanxiaojing@caas.cn (X.Y.)

**Keywords:** *Xanthomonas oryzae* pv. *oryzae* (*Xoo*), T3SS, small molecule inhibitors, virulence factors

## Abstract

The initiative strategy for the development of novel anti-microbial agents usually uses the virulence factors of bacteria as a target, without affecting their growth and survival. The type III secretion system (T3SS), one of the essential virulence factors in most Gram-negative pathogenic bacteria because of its highly conserved construct, has been regarded as an effective target that developed new anti-microbial drugs. *Xanthomonas oryzae* pv. *oryzae* (*Xoo*) causes leaf blight diseases and is one of the most important pathogens on rice. To find potential anti-virulence agents against this pathogen, a number of natural compounds were screened for their effects on the T3SS of *Xoo*. Three of 34 compounds significantly inhibited the promoter activity of the harpin gene, *hpa1*, and were further checked for their impact on bacterial growth and on the hypersensitive response (HR) caused by *Xoo* on non-host tobacco plants. The results indicated that treatment of *Xoo* with CZ-1, CZ-4 and CZ-9 resulted in an obviously attenuated HR without affecting bacterial growth and survival. Moreover, quantitative reverse transcription-polymerase chain reaction (qRT-PCR) analysis showed that the expression of the *Xoo* T3SS was suppressed by treatment with the three inhibitors. The mRNA levels of representative genes in the hypersensitive response and pathogenicity (*hrp*) cluster, as well as the regulatory genes *hrpG* and *hrpX*, were reduced. Finally, the in vivo test demonstrated that the compounds could reduce the disease symptoms of *Xoo* on the rice cultivar (*Oryza sativa*) IR24.

## 1. Introduction

The conventional methods for phytopathogenic bacteria control still rely on antibiotics, which affect the essential processes for bacterial growth and survival, and a strong resistance to antibiotics has since evolved [1,2]. An alternative approach is to find new agents that target bacterial virulence factors, rather than inhibit their essential processes of growth and survival [1,3]. The type III secretion system (T3SS) has three crucial traits that are highly conserved and are a critical virulence factor in most Gram-negative bacteria and are unnecessary for bacterial survival in vitro [4,5]. Thus, the type III secretion system is a great target for screening and developing novel anti-microbial drugs [6,7]. Until now, several different kinds of small molecules have been identified as T3SS inhibitors against a range of pathogens, including *Escherichia*, *Yersinia*, *Salmonella* and *Erwinia* species [8,9,10,11]. These inhibitors have an effect on the components of the T3SS apparatus directly [10,12], or function by regulating T3SS gene expression [11,13], or through some indirect interactions [9,12].

*Xanthomonas oryzae* pv. *oryzae* (*Xoo*) leads to bacterial leaf blight disease, one of the most devastating diseases in rice worldwide [14], resulting in severe yield losses, especially in Asia and Africa [15]. Like many other Gram-negative phytopathogenic bacteria, *Xoo* injects and delivers effector proteins into host cells through a T3SS, which is encoded by the gene locus of hypersensitive response and pathogenicity (*hrp*) [16,17]. The T3SS apparatus play a key role in conferring pathogenicity on the host, which can trigger a hypersensitive response (HR) on non-host or resistant plants [18]. More than 20 genes form the core operon on several transcriptional units, which contain *hrp*, *hpa* (*hrp* associated) and *hrc* (*hrp*-conserved) genes [16,17]. There are two types of effector in *X. oryzae*: TAL (transcription activator-like) and non-TAL effectors [19,20], which often determine the consequence of interactions between bacteria and different hosts. *Hrp* gene expression is tightly regulated, and is induced in planta or in a prepared medium designed to mimic in planta conditions and suppressed in nutrient-rich medium [21,22]. Two types of *hrp* genes have been classified, and the *hrp* genes in *Xanthomonas* spp. and *Ralstonia solanacearum* are *hrp* group II, which is different from group I in *Pseudomonas syringae* and *E. amylovora* [18,22]. The expression of *hrp* genes in group II is activated by two key known regulatory genes, *hrpG* and *hrpX*, which are located spatially away from the *hrp* gene cluster [23,24]. While in group I, the expression of *hrp* genes is regulated by alternative sigma factor HrpL [25,26].

We know that HrpG belongs to the OmpR family of two-component signal transduction systems (TCS), which is one of the response regulators to regulate the expression of *hrpX* positively [24]. HrpX is a regulator of AraC family, and mainly activates the transcription of other *hrp* genes (*hrpB* to *hrpF*), and together they encode T3 effectors [23]. In the HrpG regulon, most genes are regulated by HrpX, because the HrpX interacts with the plant inducible promoter (PIP)-box, which has a *cis*-element within the promoter region of *hrp* genes and is present in the promoter of many T3 effectors [23,27].

In this article, a number of natural compounds were screened for their effectiveness on T3SS of *Xoo*. Three of them were selected for further analysis without killing bacteria. The mechanisms of the inhibitors were evaluated by examining the effects on expression of representative *hrp* genes. In planta assays indicated that the inhibitors could weaken the symptoms on rice caused by *Xoo*.

## 2. Results and Discussion

### 2.1. Screening of Inhibitors Which Affect the T3SS of Xoo

**Figure 1:**
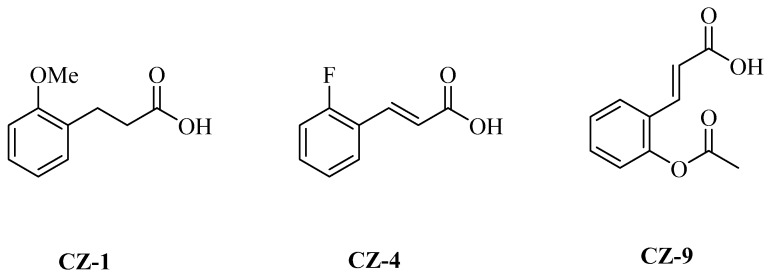
Chemical structures of three bioactive compounds.

To find potential inhibitors that inhibit the expression of T3SS in *Xoo*, a number of 34 natural compounds (Table 1 and Table S1) were tested for their effects on the promoter activity of the *hpa1* gene, which is induced in the *hrp*-inducing medium XOM2 [28,29]. *Hpa1* was encoded by a harpin protein in *Xoo* [28], and its expression controlled by the regulatory protein HrpX. A reporter plasmid, pPhpa1, and the promoter region of *hpa1* (Figure S1) were constructed into the promoter-probe vector pPROBE-AT [30], which has a promoterless green fluorescence protein (GFP) reporter gene. The pPhpa1 was transformed into the *Xoo* PXO99^A^ strain, and then grown in XOM2 and treated with tested compounds at 200 μM for 15 h before the promoter activity of *hpa1* was examined. The highly efficient fluorescence-activated cell sorting (FACS) system was applied to check for alterations in *hpa1* promoter activity. The mean fluorescence intensity (MFI) is recorded in Table 1, which represents the promoter activity of *hpa1*. The ratio of MFI after treatment by the tested compounds compared to that of the dimethylsulfoxide (DMSO) control was calculated, and the result listed in Table 1, indicated by %DMSO. The results showed that three compounds (Figure 1) inhibited the *hpa1* promoter activity by at least 60% (Table 1), which indicated these compounds repressed *hpa1* promoter activity significantly compared with the solvent control.

Rice is the staple food for the half population of the world. At the same time, rice is vulnerable to pathogen infection, which not only leads to devastating diseases, but also brings severe yield losses. Bacterial leaf blight and leaf streak diseases mainly caused by the two pathovars of *X. oryzae* are the most important bacterial diseases around the world, especially in Asia and Africa [15]. Here, we report that phenolic compounds CZ-1, CZ-4 and CZ-9, suppressed the disease symptoms of *Xoo* and *Xoc* on rice by specifically inhibiting the function of T3SS.

The compounds were identified by screening from a small library of natural phenolics and their derivatives, some of which have previously been shown to affect the T3SS gene expression of several bacterial pathogens, like *D. dadantii* [30,31], *P. aeruginosa* [32] and *E. amylovora* [33]. Marshall et al. used a whole-cell-based high-throughput screening (HTS) approach to identify pneumonia T3SS inhibitors from compound libraries [6]. Here, the *Xoo* strain PXO99^A^, harboring a reporter plasmid with a *gfp* gene transcriptionally fused to the *hpa1* promoter, was constructed for screening. Eight of 9 compounds showed significant inhibitory effects on *hap1* promoter activity, as shown in Table 1. This efficiency is much higher than that of HTS using large libraries containing thousands of small natural or synthetic molecules [7,8].

### 2.2. Measurement of Growth Curve

**Figure 2:**
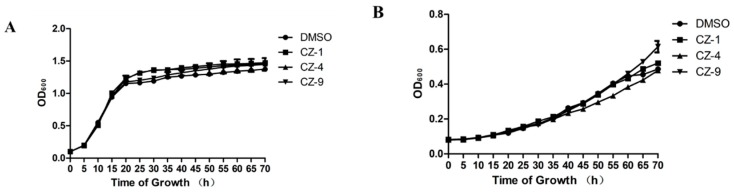
Growth rates of PXO99^A^ in rich medium M210 (**A**) and plant-mimicking medium XOM2 (**B**) supplemented with 200 μM compounds. The optical density at 600 nm (OD_600_) of the culture suspensions was measured every 1 h during a 72 h period. Three independent tests were performed with similar results.

The reason we utilize the target virulence factors of bacteria to screen is because they do not affect their growth. So, the influence of the selected compounds on bacterial growth was investigated at two different stages, and both *hrp*-inducing medium (XOM2, 0.5% sucrose was supplemented to support bacterial growth) and rich medium (M210) were included. The growth rate of *Xoo* was measured in a period of 72 h and the concentration of CZ-1, CZ-4 and CZ-9 were added to the media at 200 μM. According to the growth curve, the compounds had no obvious inhibition on the bacterial growth at different time points. Bacteria with the addition of compounds CZ-1, CZ-4 and CZ-9 compared to the solvent control did not show significant changes at different time points, indicating that these compounds did not affect bacterial growth, as shown in Figure 2. On the contrary, the other eighteen title compounds affected bacterial growth to various degrees (Figure S2). As the aim is to look for compounds that suppressed bacterial virulence, bacterial growth or survival cannot be affected. From this, we focused on CZ-1, CZ-4 and CZ-9 for the remainder of the study. To be consistent, we used a final concentration of 200 μM of each compound in all other assays.

### 2.3. Three Compounds Suppress the HR Caused by Xoo in Tobacco

**Figure 3:**
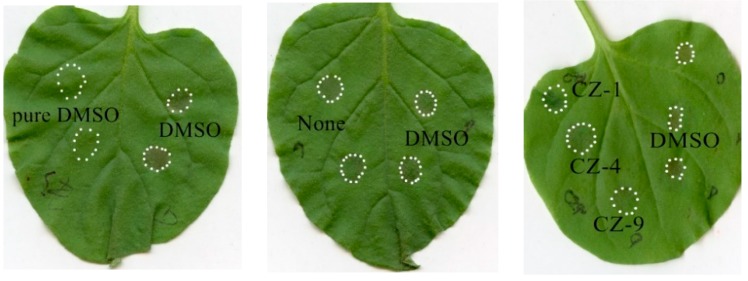
Effects of various compounds on the hypersensitive response (HR) induced by *Xanthomonas oryzae* pv. *oryzae* (*Xoo*) on *Nicotiana benthamiana*. Cell suspensions of wild-type *Xoo* PXO99^A^ (wild-type, WT) at an optical density of 600 nm (OD_600_) of 0.5 were incubated with dimethylsulfoxide (DMSO) or each compound at a concentration of 200 μM for 2 h before infiltration into tobacco leaves. Photographs were taken 24 h after infiltration. At least three independent experiments were performed with similar results.

The HR-inducing ability can give direct evidence as to whether T3SS is active in the bacteria. Because Hpa1 has previously been confirmed to be a T3SS-secreted harpin protein in both *Xoo* and *Xoc* [16,28], the HR can be induced by a harpin protein. Therefore, the HR-inducing ability of *Xoo* on tobacco was tested to further determine if the function of the three inhibitors interfered with the T3SS in *Xoo*. Infiltration of *Xoo* bacterial germs into non-host tobacco leaves can induce HR, as shown in Figure 3. Before infiltrated into tobacco leaves, the bacterial germ suspensions were incubated with the tested compound or DMSO for 2 h. When the concentration of the inhibitors increased to 200 μM, CZ-1, CZ-4 and CZ-9 caused a complete loss of HR (Figure 3), suggesting that these compounds could effectively suppress the function of the *Xoo* T3SS.

### 2.4. Expression of Representative hrp/hrc Genes is Suppressed by Inhibitors CZ-1, CZ-4 and CZ-9

**Figure 4:**
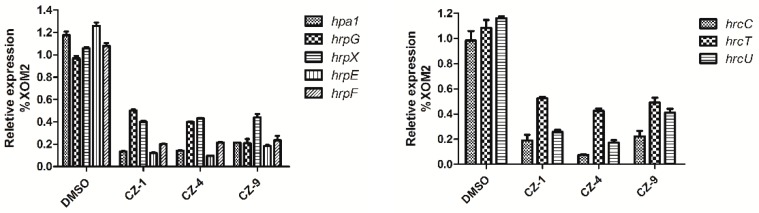
The effect of the three T3SS inhibitors on the expression of representative genes in the *hrp* cluster.

The above results demonstrate that CZ-1, CZ-4 and CZ-9 repress the *Xoo* T3SS, but do not influence the bacterial growth, suggesting that the three compounds might target the regulatory pathway specifically for affecting T3SS gene expression. Therefore, qRT-PCR was carried out to check the effect of these compounds on the expression levels of T3SS genes. In Table 1, the data showed that CZ-1, CZ-4 and CZ-9 reduced the promoter activity of *hpa1* by at least 60%. The qRT-PCR assays in Figure 4 showed that the mRNA level of *hpa1* was decreased around 80% in *Xoo* germs treated with these inhibitors (CZ-1: 87%, CZ-4: 86%, CZ-9: 79%, respectively), which is consistent with the promoter assay in Table 1. Then, the expression levels of other *hrp*/*hrc* genes were examined, including two *hrp* genes (*hrpE* and *hrpF* encoding the *hrp* pilus protein and a putative translocon protein respectively) and three *hrc* genes (*hrcC, hrcT* and *hrcU* encoding the outer-membrane secretion and export apparatus genes respectively) [34]. The results in Figure 4 showed that, compared with DMSO control, the mRNA levels of the tested *hrp*/*hrc* genes were reduced obviously when mixed with the three inhibitors CZ-1, CZ-4 and CZ-9.

**Figure 5:**
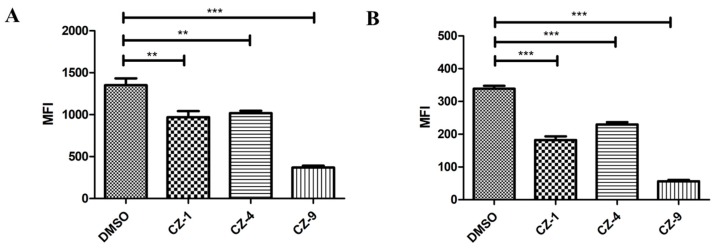
Promoter activities of *hrpG* and *hrpX* in *Xoo* grown in XOM2 supplement with 200 μM of CZ-1, CZ-4, and CZ-9 for 15 h, measured by fluorescence-activated cell sorting (FACS). At least three independent tests were performed with similar results. Asterisks indicate statistically significant differences (Student’s *t*-test) ** *p* < 0.05, *** *p* < 0.01.

Our results indicated that CZ-1, CZ-4 and CZ-9 suppressed *hrp*/*hrc* gene expression probably through the major regulatory proteins HrpG and HrpX, as shown in Figure 5. Of course, more work needs to be performed in the future to fully illustrate the exact mechanism. As a result of their possible positive impact on agriculture, it is important to understand the functional mechanism of these T3SS inhibitors.

### 2.5. The three Inhibitors Affect the Expression of Regulatory Genes hrpG and hrpX

Given that the expression of *Xanthomonas* T3SS is regulated by a cascade consisting of HrpG and HrpX [5]. It was necessary to evaluate whether the expression of *hrpG* and *hrpX* was affected due to a reduction in mRNA levels in the representative *hpa*/*hrp*/*hrc* genes in *Xoo* treated with the inhibitors. The mRNA levels of *hrpG* and *hrpX* were investigated when *Xoo* germs were mixed with the inhibitors, and the results indicated that the mRNA level of *hrpX* was reduced by about 60% (CZ-1: 60%; CZ-4: 57%; CZ-9: 56%, respectively) in comparison with the DMSO control. Meanwhile, the mRNA level of *hrpG* was also reduced more than 50% when it contained CZ-1, CZ-4 and CZ-9 (Figure 5). Among them, CZ-9 showed the best activity and made the mRNA level of *hrpG* reduced by approximately 80% compared with the DMSO control. These results indicated the inhibitory effect of CZ-1, CZ-4 and CZ-9 on T3SS gene expression of *Xoo* by the HrpG–HrpX regulatory cascade.

### 2.6. The promoters of hrcT Respond Differentially to Inhibitors CZ-1, CZ-4 and CZ-9

**Figure 6:**
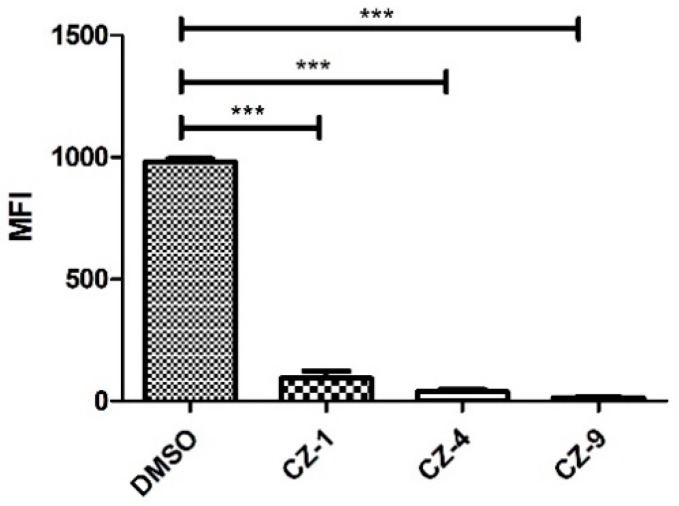
Promoter activities of *hrcT* in *Xoo* grown in XOM2 supplement with 200 μM of CZ-1, CZ-4, and CZ-9 for 15 h, measured by fluorescence-activated cell sorting (FACS). At least three independent tests were performed with similar results. Asterisks indicate statistically significant differences (Student’s *t*-test) *** *p* < 0.01.

HrpX is the key transcriptional regulator of many downstream genes in the *hrp* cluster and distinguishes the PIP-boxes (TTCGC-N15-TTCGC) in the promoter region, such as that of *hpa1* (Figure S1). To best exhibit the effect of the three inhibitors on the transcription of T3SS genes in *Xoo*, the promoter for the *hrcT* operon (named PhrcT) contained a perfect PIP-box and was used to investigate the impact of CZ-1, CZ-4 and CZ-9 on the activity of PhrcT by FACS assays. The results in Figure 6 showed that the activity of PhrcT decreased by at least 90% after treatment with CZ-1 (90%), CZ-4 (96%) and CZ-9 (98%) in comparison with the DMSO control (DMSO%). Their effects coincide with the statistical analysis of the *hpa1* promoter, which demonstrated that the targets of these inhibitors were the promoters with a PIP-box. The results also supported that HrpX played a key role in the regulation of the inhibitory function.

In *Xoc*, PhrcC was positively regulated by HrpG, but not HrpX [35]. The activity of PhrcT was suppressed by the three T3SS inhibitors, suggesting that the regulatory role of HrpX might be indispensable for the function of these inhibitors. We speculated that these inhibitors may exert their effects upstream of HrpG, as the expression of *hrpG* and *hrpX* was affected (Figure 5). Presumably, phosphorylated HrpG activated the expression and production of HrpX, and HrpX regulated the downstream genes [5]. However, the signaling transduction upstream of HrpG remains elusive. Recently, a putative histidine kinase of HrpG has been reported in *X. campestris* pv. *campestris* [35]. In addition, the RNA-binding protein RsmA has been shown to positively regulate the T3SS by stabilizing HrpG mRNA in *X. citri* ssp. *citri* [36]. It will be of great interest to study whether these components play similar roles in regulating the T3SS in *X. oryzae*, and whether they mediate the inhibitory effects of these compounds on the T3SS. By comparison with previous screening results of these phenolic compounds in other bacteria, we observed some differences in their activities. For example, TS006 (OCA, Table S1), the inhibitor of the *Xoo* T3SS [2], which has also been shown to inhibit the T3SS of *E. amylovora* [33], was initially identified to induce T3SS gene expression in *D. dadantii* [30,31]. In contrast, TS004 (PCA), a strong inhibitor of the T3SS in *D. dadantii* and *E. amylovora*, did not show significant alteration of *hpa1* promoter activity in *Xoo*. In *D. dadantii*, PCA inhibited the T3SS gene expression through the HrpX/HrpY TCS [30], which is a pathway that does not exist in *Xoo*. Therefore, different T3SS regulatory pathways might be a major reason for the distinctive activities detected for these compounds. Given the possibility that the T3SS inhibitors might also affect the expression of other virulence factors, it was important to observe the behavior of these types of T3SS inhibitor. In *Salmonella*, when a compound was used to inhibit the secretion of the T3 substrate SipA, a significantly increased amount of the flagellin protein FliC was detected [8]. This might be a result of cross-talk between virulence factors in bacteria, which would warrant further investigation.

### 2.7. CZ-1, CZ-4 and CZ-9 Suppress the Water Soaking and Disease Symptoms of Xoo on Rice

**Figure 7:**
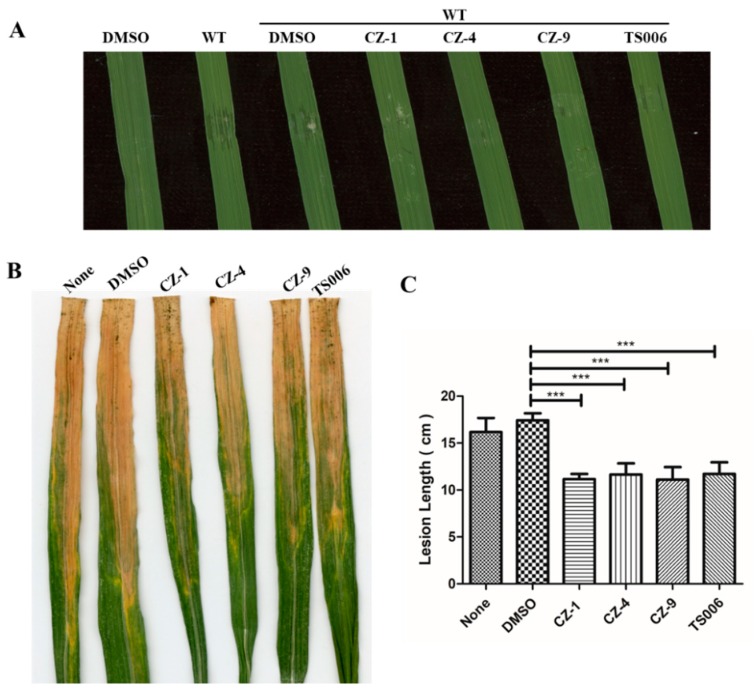
(**A**) The effect of CZ-1, CZ-4, CZ-9 and TS006 on water-soaking symptoms. From left to right, provide pure DMSO, WT, DMSO + WT, CZ-1, CZ-4, CZ-9 and TS006 (**B**,**C**) lesion lengths of *Xoo* PXO99^A^ on adult plants of rice cultivar IR24 were reduced after pretreatment with CZ-1, CZ-4, CZ-9 and TS006. Photographs were taken 14 days after infiltration. At least three independent tests were performed with similar results. Asterisks indicate statistically significant differences (Student’s *t*-test). *** *p* < 0.01.

The final purpose of this work was to confirm that the selected inhibitors could suppress the virulence phenotypes of *Xoo* in planta. The seedlings of susceptible rice (cultivar IR24) were used to evaluate the symptoms of water-soaked lesions induced by *Xoo* PXO99^A^ after infiltration of bacterial germ into the leaves. The results in Figure 7 showed that both the water soaking symptoms on seedlings (Figure 7A), and the yellowish disease symptoms on adult IR24 plants (Figure 7B), were reduced and weakened to various extents by treatment of the bacterial germs with CZ-1, CZ-4 and CZ-9. Moreover, the lesion lengths were significantly reduced by treatment of CZ-1 (11.8 cm), CZ-4 (11.6 cm) and CZ-9 (11.1 cm) in comparison with DMSO (15.7 cm) (Figure 7C).

Water soaking is a symptom specifically induced by the AvrBs3/PthA family of effectors in *Xanthomonads*, then they were renamed as TAL (transcription activator-like) effectors [20,37]. In *Xoo* strain PXO99^A^ there exist multiple TAL effectors, which confer gene-specific resistant to host plants [20]. After inoculation on the susceptible rice cultivar IR24, owing to lack of corresponding *R* genes, the PXO99^A^ strain caused strong water-soaked lesions [37] (Figure 7A). After performing a treatment protocol with these compounds, we found that water soaking phenotypes on rice were almost completely abolished (Figure 7).

**Figure 8:**
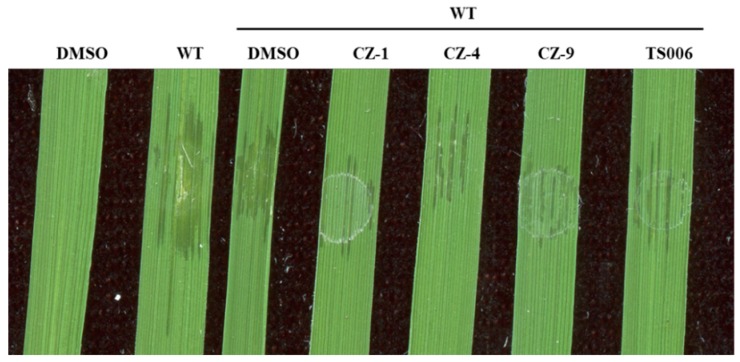
The impact of CZ-1, CZ-4, CZ-9 and TS006 on disease symptoms caused by infiltration of *Xoc* RS105 (wild-type, WT) in IR24 seedlings. Photographs were taken 3 days after infiltration. At least three independent tests were performed with similar results.

Although these T3SS inhibitors were screened using *Xoo* as the reporter strain, it was not surprising to observe their functions in *Xoc*, as the major T3SS regulatory pathways are very similar between *Xoo* and *Xoc* [16,17]. With regard to why the inhibitors were more efficient in suppressing the virulence of *Xoc*, (Figure 8) different infection methods between the two pathovars might be a possible reason. In summary, we have demonstrated the inhibitory effect of phenolic compounds CZ-1, CZ-4, CZ-9 and TS006 on the T3SS of *Xoo* both in vitro and in planta. Furthermore, these compounds have been proven to be effective in suppressing the disease symptoms caused by *Xoo* and *Xoc* on rice leaves. These findings are important because they may provide potential anti-virulence drugs, which might be used to prevent infection in agricultural production in the future.

## 3. Materials and Methods

### 3.1. Bacterial Strains, Plasmids and Growth Conditions

The plasmids and bacterial strains used in this study were listed in Table 2. *Xanthomonas oryzae* pv. *oryzae* (*Xoo*) PXO99^A^ strain and the derived strains were grown in M210 medium (0.5% sucrose, 0.8% casein enzymatic hydrolysate, 0.4% yeast extract, 1.2 mM MgSO_4_·7 H_2_O, 17.2 mM K_2_HPO_4_) or on PSA plates. *Escherichia coli* was cultivated in Luria Bertani (LB) medium at 37 °C. XOM2 medium (670 mM l-methionine, 0.18% d-(+) xylose, 10 mM sodium l-(+) glutamate, 40 mM MnSO_4_, 14.7 mM KH_2_PO_4_, 5 mM MgCl_2_ and 240 mM Fe(III)-EDTA, with the pH adjusted to 6.5 with KOH) was applied for *hrp*-inducing conditions. Ampicillin (Ap) and cephalexin (Cp) were added to media at the final concentrations of 100 and 25 μg/mL.

### 3.2. Sources of the Screened Compounds

All the tested compounds were procured from commercial sources from Macklin (Shanghai, China, Table S1). All the compounds were dissolved in DMSO.

### 3.3. Flow Cytometry Analysis

PXO99^A^ containing the pPhpa1 and promoterless pPROBE-AT was cultured in M210 overnight, and then transferred to XOM2 and added to 200 μM of the tested compounds. Samples were diluted to the appropriate concentration with 1× phosphate-buffered saline (PBS) at 15 h after inoculation. The promoter activity of *hpa1* was checked by measuring the GFP intensity using a FACS-Caliber flow cytometer (CytoFLEX, Brea, CA, USA) [32]. DMSO was used as a negative control. Three independent experiments were performed, and three replicates were used each time. Similar methods were applied to analyze the promoter activities of *hrpG*, *hrpX* and *hrcT*.

### 3.4. RNA Extraction and qPCR Analysis

*Xoo* germ were grown in M210 overnight at 28 °C, subcultured to XOM2 at 600 nm (OD_600_) of 0.6, and added into DMSO or 200 μM of tested compounds and shake culture at 28 °C for 15 h. The total RNA was extracted from the collected cells with an RNAprep Pure Bacteria Kit (Promega, USA). RNA degradation and contamination were checked on 1% agarose gels and RNA concentration and purity were monitored using the Nanovue UV-Vs spectrophotometer (GE Healthcare Bio-Science, Sweden). cDNA was synthesized using an HiScriptII Q RT SuperMix Kit (Tiangen, Beijing, China). The cDNA levels were quantified by real-time PCR using a SYBR Green Master Mix (Thermo Scientific, MA, USA). The relative levels of gene expression were analyzed by the 2^−∆∆*C*t^ method [38]. The DNA gyrase subunit B (gyrB) gene was used as the internal control (Table S2) [21]. Expression values are the means of three biological repeats in each experiment. The Student’s *t*-test was used for statistical analysis.

### 3.5. Measurement of the Growth Rate

*Xoo* were cultured overnight in M210 at 28 °C. The bacterial suspensions were adjusted to OD_600_ ≈ 1.0 using relevant medium, then transferred into M210 or XOM2 (plus 0.5% sucrose) medium containing 200 μM of tested compounds or DMSO, starting at an OD_600_ of 0.08. Setting parameters of growth curve instrument, the growth rates were recorded every 1 h during the 72 h period using a Bioscreen (Bioscreen, Finland). Growth of *Xoo* PXO99^A^ in the presence of the compound was compared with the growth of control (*Xoo* PXO99^A^ without DMSO). Three independent experiments were performed, and three replicates were used in each experiment. The Student’s *t*-test was used for statistical analysis.

### 3.6. HR Assay

*Xoo* cells were grown in M210 overnight at 28 °C, and then suspended in sterile distilled water. The bacterial suspensions were adjusted to OD_600_ of 0.5. *Nicotiana benthamiana* plants were used for HR assays. To the adult plants, each flag leave was inoculated with PXO99^A^ suspensions with 15–20 independent individuals, respectively. Plants were scored and the symptoms were checked at 3 days post-inoculation (dpi) in seedlings, and at 14 dpi for lesion lengths (the length from the tip to the leading edge of the grayish symptom) in adult rice plants. Treatments were compared by using the Tukey test for analysis after ANOVA. The mean lesion length of ten leaves was used for each treatment. During the experiments, plants were maintained in a greenhouse at 25 °C (16 h of light and 8 h of darkness).

### 3.7. Statistical Analyses

Statistical analyses were performed using GraphPad Prism 6.0 software. Descriptions of the tests used are introduced in detail in figure legends.

## 4. Conclusions

A number of 34 natural compounds were screened for their effects on the promoter activity of the *hpa1* gene, which is induced in the *hrp*-inducing medium XOM2. The bioassay showed that three of the compounds CZ-1, CZ-4 and CZ-9 inhibited the promoter activity of a harpin gene *hpa1* significantly, and led to significantly attenuated hypersensitive response without affecting bacterial growth or survival. Further mechanism study demonstrated that the mRNA levels of representative genes in the *hrp* (hypersensitive response and pathogenicity) cluster, as well as the regulatory genes *hrpG* and *hrpX*, were reduced by compounds CZ-1, CZ-4 and CZ-9 suggesting that the expression of the *Xoo* T3SS was suppressed by treatment with the three compounds. Finally, the in vivo test showed that the compounds could reduce the disease symptoms of *Xoo* on the rice cultivar (*Oryza sativa*) IR24. These findings afford potential anti-virulence agents, which might be applied to inhibit infection in agricultural production.

## Figures and Tables

**Table 1:** Screening for inhibitors of *Xoo* T3SS by fluorescence-activated cell sorting assays.

Compound	Avg MFI ± SD ^a^	DMSO% ^b^	Inhibition Rate % (100%—DMSO%)
DMSO	12754.03 ± 534.05		
CZ-1	1754.60 ± 14.71 *	13.76	86.24
CZ-2	3781.20 ± 125.84 *	29.65	70.35
CZ-3	3951.57 ± 408.15 *	30.98	69.02
CZ-4	1849.17 ± 87.95 *	14.50	85.50
CZ-5	1049.47 ± 43.36 *	8.23	91.77
CZ-6	11181.52 ± 258.22	87.67	12.33
CZ-7	2842.00 ± 100.47 *	22.28	77.72
CZ-8	705.25 ± 185.00 *	5.53	94.47
CZ-9	726.43 ± 11.25 *	5.70	94.3
DMSO	17185.37 ± 2439.44		
FP1	3482.30 ± 569.36 *	20.26	79.74
FP2	25248.53 ± 2983.44	146.92	−46.92
FP3	21246.93 ± 801.74	123.63	−23.63
FP4	6747.17 ± 1114.18 *	39.26	60.74
FP5	19622.43 ± 676.17	114.18	−14.18
FP6	13012.07 ± 606.73	75.72	24.28
FP7	20247.13 ± 5916.35	117.82	−17.82
FP8	112.57 ± 2.54 *	0.66	99.34
FP9	19572.67 ± 2290.71	113.89	−13.89
FP10	17977.20 ± 2973.48	104.61	−4.61
FP11	22372.47 ± 1607.45	130.18	−30.18
FP12	6355.20 ± 683.35 *	36.98	63.02
FP13	6942.47 ± 1340.16	40.40	59.60
FP15	9733.60 ± 1209.31	56.64	43.36
DMSO	4378.07 ± 400.82		
HF1	3646.93 ± 961.61	83.30	16.70
HF6	181.63 ± 3.57 *	4.15	95.85
HF8	3715.17 ± 347.52	84.86	15.14
HF11	182.40 ± 8.07 *	4.17	95.83
HF12	1022.33 ± 148.35 *	23.35	76.65
HF13	1466.87 ± 52.55 *	33.50	66.50
HF15	190.20 ± 16.62 *	4.34	95.66
HF17	161.10 ± 3.43 *	3.68	96.32
HF18	1432.37 ± 180.47 *	32.72	67.28
HF19	1852.33 ± 278.62	42.31	57.69
FP31	5504.43 ± 625.60	125.73	−25.73

^a^ Green fluorescent protein (GFP) mean fluorescence intensity (MFI) was detected for gated populations of bacterial cells by flow cytometry. Values are representative of at least three independent experiments, and three replicates were used for each experiment. Asterisks indicate statistically significant differences in MFI between bacterial cells grown in XOM2 with DMSO and XOM2 supplemented with 200 μM of each compound (Student’s t-test). * *p* < 0.05. ^b^ %DMSO was used to represent the relative promoter activity of *hpa1* in *Xoo* cells grown in XOM2 supplemented with 200 μM of each compound in comparison with that in XOM2 with DMSO only, which was calculated by the formula: %DMSO = 100 × MFI (XOM2 with compounds)/MFI (XOM2 with DMSO).

**Table 2:** Strains and plasmids used in this study.

Strains and Plasmids	Relevant Characteristics	Reference or Source
**Strains**		
*Xanthomonas oryzae* pv. *oryzae*		
PXO99^A^	Wild-type strain, Philippine race 6, Cpr	Dr. Chenyang He
*Xanthomonas oryzae* pv. *oryzicola*		
RS105 Wild-type	Chinese race 2	Dr. Fengquan Liu
**Plasmids**		
pPROBE-AT	Promoter-probe vector, Apr	
pPhpa1 pProbe-AT	Derivative with PCR fragment containing *hpa1* promoter region, Apr	Dr. Chenyang He
pPhrcT pProbe-AT	Derivative with PCR fragment containing *hrcT* promoter region, Apr	Dr. Chenyang He
pPhrpG pProbe-AT	Derivative with PCR fragment containing *hrpG* promoter region, Apr	Dr. Chenyang He
pPhrpX pProbe-AT	Derivative with PCR fragment containing *hrpX* promoter region, Apr	Dr. Chenyang He

Apr, ampicillin resistance; Cpr, cephalexin resistance.

## Supplementary Materials

Supplementary materials can be found at https://www.mdpi.com/1422-0067/20/4/971/s1.
