# Supplementary material for: Small Molecule Inhibitors Specifically Targeting the Type III Secretion System of Xanthomonas oryzae on Rice
Source: Int J Mol Sci. 2019 Feb 23;20(4):971. doi: 10.3390/ijms20040971 (PMC6412923; doi:10.3390/ijms20040971)
Supplement: Supplementary file 1 [file ijms-20-00971-s001.pdf]

International Journal of *Molecular Sciences*

MDPI

Supplementary Materials

Phpa1-F
CCACTTAACGGGCAAGCGAAAAAAGCTTTTCTCAACAACGCCCCGCGGATTTGTTATCGA 60
PIP-box
TTCTAAAACATTTTTTCACTTGCCCTC**TTCGC**GCGTACAAGCGCAAT**TTCGC**AAAATTTC 120

TGGCGATGATGGGCTTCCGCTTCTACTGTTTGATCGGGGCGCAAAACGCGCCTCGCAGCC 180
Phpa1-R
ACCGCTGTCTAGGCACGGCTGTTGATACTAAAGACACATACCATTTA**ATCAGAGAGGAAT** 240
Start codon
CGTCACGATGAATTCTTTGAACACACAATTCGGCGGCAGCACGTCCAACCTTCAGGTTGG 300

CCCAAGCCAGGACACAACGTTCGGTTCGAACCAGGGCGGCAACCAGGGCATCTCGGAAAA 360

GCAACTGGACCAGTTGCTGTGCCAGCTCATCTCGGCCCTGCTTCAGTCGAGCAAAAATGC 420

TGAGGAGGGTAAGGGTCAGGGTGGCGATAATGGCGGTGGCCAGGGCGGCAATTCGCAGCA 480

GGCTGGGCAGCAGAATGGCCCCTCGCCATTCACCCAGATGCTGATGCATATCGTCGGAGA 540

GATTCTCCAGGCGCAGAATGGTGGTGGTGCTGGTGGCGGCGGGTTCGGCGGCGGGTTCGG 600

CGGTGACTTTAGTGGCGACCTCGGCCTCGGCACCAACCTCTCGAGCGACAGCGCATCGAT 660
Stop codon
GCAG**TAA** 667

Figure S1. DNA sequence of *hpa1* gene and its promoter region. Sequences for a PIP box, start codon, stop codon and the primers used for amplification of the promoter region are indicated as bold fonts.

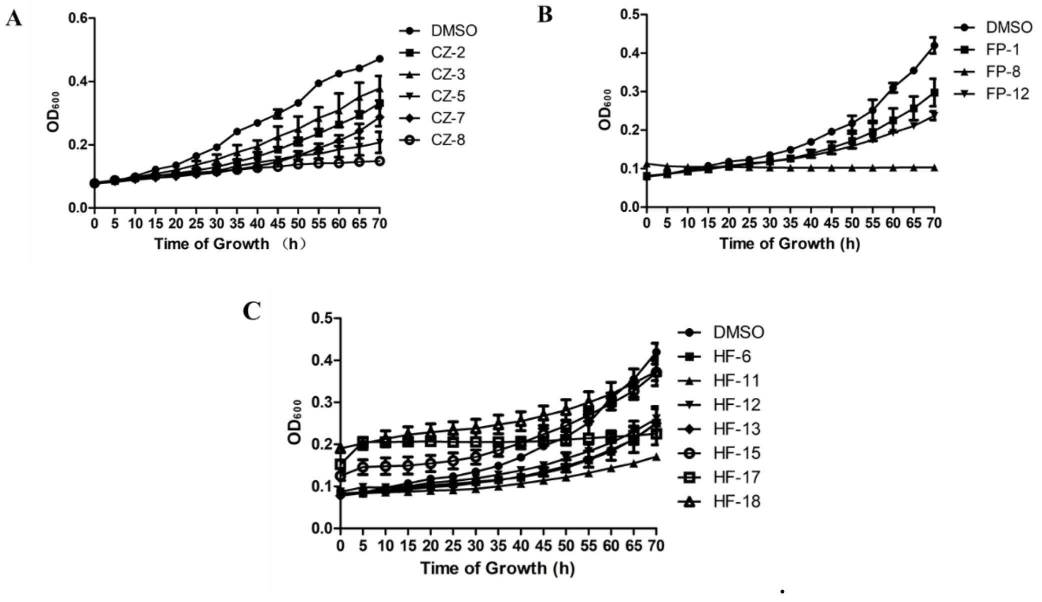

Figure S2. Effects of various compounds on bacterial survival, Growth rates of PXO99^{A} in plant-mimicking medium XOM2 supplemented with 200 μmol/L compounds. The optical density at 600 nm (OD_{600}) of the culture suspensions was measured every 1 h during the 72 h period. Three independent tests were performed with similar results.

A

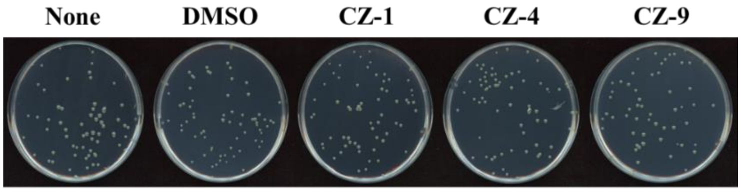

B

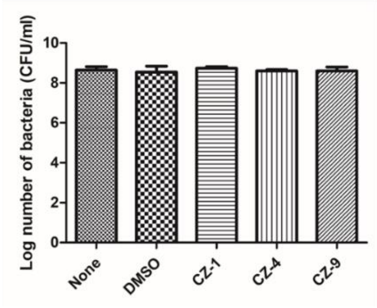

Figure S3. Effects of various compounds on bacterial survival. Bacterial cells were incubated with DMSO or various compounds at a concentration of 200 μM for 2 h at 28 °C before being plating on PSA plate after serial dilutions. Three independent tests were performed with similar results.

Table S1. The compounds in this study.

No.	Compounds	Structure	Name
1	CZ-1	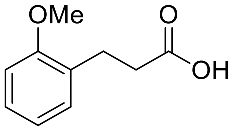	2-methoxybenzene propanoic acid
2	CZ-2	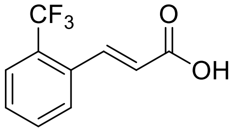	3-[2-(trifluoromethyl)phenyl]-2 -propenoic acid
3	CZ-3	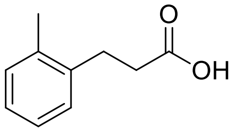	2-methylbenzene propanoic acid
4	CZ-4	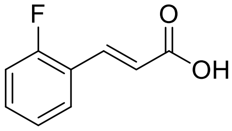	3-(2-fluorophenyl)-2-propenoic acid
5	CZ-5	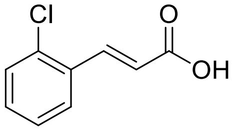	3-(2-chlorophenyl)-2-propenoic acid
6	CZ-6	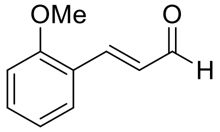	3-(2-methoxyphenyl)-2-propenal
7	CZ-7	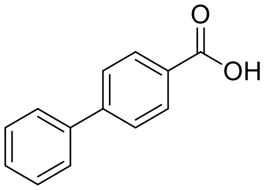	[1,1′-biphenyl]-4-carboxylic acid

8	CZ-8	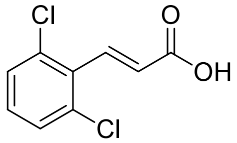	3-(2,6-dichlorophenyl)-2-propenoic acid
9	CZ-9	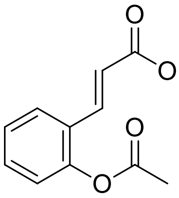	3-[2-(acetyloxy)phenyl]-2-propenoic acid
10	FP-1	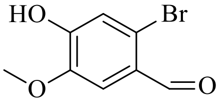	6-bromo vanillin
11	FP-2	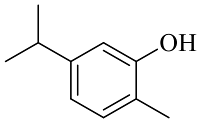	carvacrol
12	FP-3	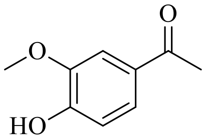	aceto vanillone
13	FP-4	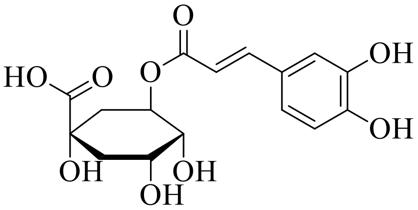	chlorogenic acid
14	FP-5	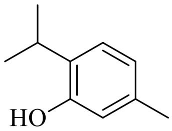	thymol
15	FP-6	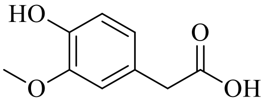	homovanillic acid
16	FP-7	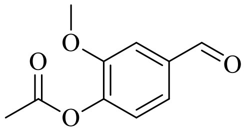	vanillin acetate
17	FP-8	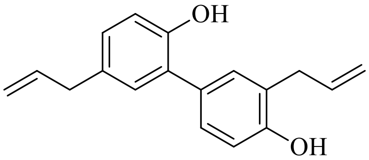	honokiol
18	FP-9	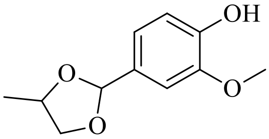	vanillin propylene glycol acetal
19	FP-10	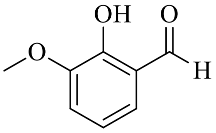	2-hydroxy-3-methoxy-benzaldehyde
20	FP-11	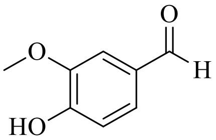	4-hydroxy-3-methoxy-benzaldehyde

21	FP-12	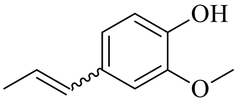	isoeugenol, mixture of *cis* and *trans*
22	FP-13	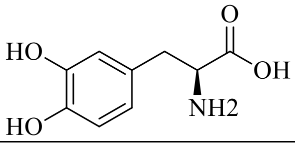	3,4-dihydroxy-L-phenylalanine
23	FP-15	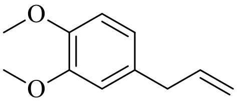	methyl eugenol
24	HF-1	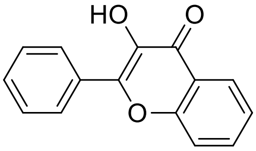	flavonol
25	HF-6	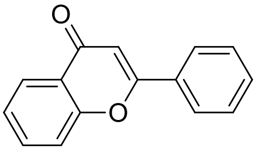	flavone
26	HF-8	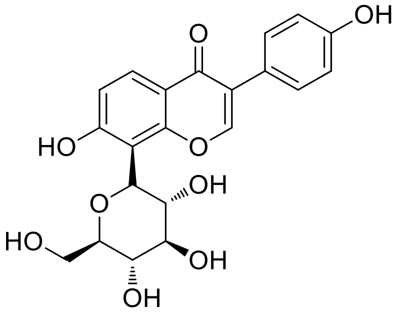	8-(*β*-D-glucopyranosyl-7-hydroxy-3-(4-hydroxyphenyl)-4*H*-1-benzopyran-4-one
27	HF-11	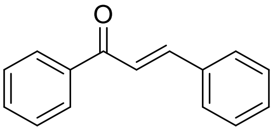	*trans*-chalcone
28	HF-12	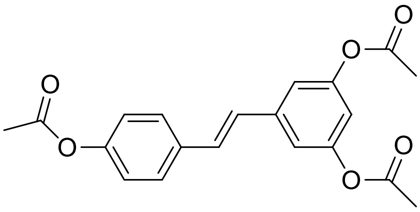	triacetyl resveratrol
29	HF-13	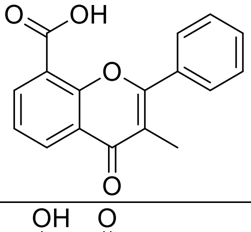	3-methylflavone-8-carboxylic acid
30	HF-15	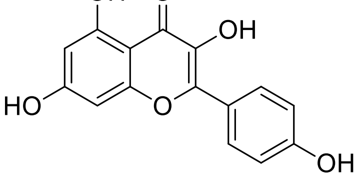	3,4′,5,7-tetrahydroxyflavone 3,5,7-trihydroxy-2-(4-hydroxyphenyl)-4*H*-1-benzopyran-4-one robigenin

31	HF-17	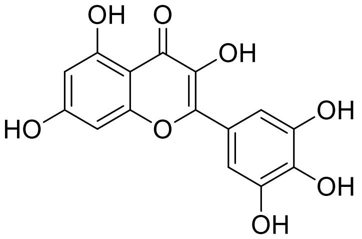	3,3′,4′,5,5′,7-hexahydroxy-flavone cannabiscetin
32	HF-18	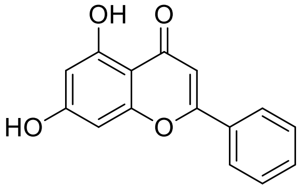	5,7-dihydroxyflavone
33	HF-19	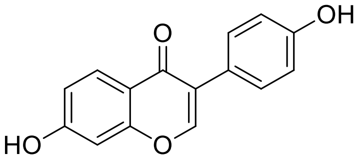	4′,7-dihydroxy-*iso*-flavone
34	FP-31	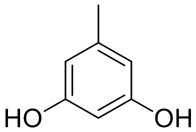	1,3-dihydroxy-5-methylbenzen e
35	TS006	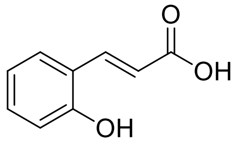	*O*-coumaric acid (OCA)

Table S2. Primers used in this study.

Primer name	Sequences (5′-3′, restriction sites underscored)	Description of amplified product
PhrpB-F	ACGCGTCGACATTTAAATGCGCCCAACTTC	
PhrpB-R	GGAATTCTTGAATCTTCTCCACACTG	234-bp promoter of $hrpB_1$
hrpG-F	TGTCCACCTGATGAACGACCCT	
hrpG-R	GGCGAATGCCGCAACGAA	*hrpG* gene fragment, 165bp
hrpX-F	AGGCACTGACCCACTTTC	
hrpX-R	ATCGGAAGCACCACTCTC	*hrpX* gene fragment, 101bp
hpa1-F	AAGCCAGGACACAACGTTCG	
hpa1-R	GAAGCAGGGCCGAGATGAG	*hpa1* gene fragment, 101bp
hrcC-F	GCGCTTTCCGGTGCGTTAC	
hrcC-R	CCCTGCTCGACCTGCTTGG	*hrcC* gene fragment, 105bp
hrcT-F	AGGGCGTGTCGCTGTTGACC	
hrcT-R	CGCGATTCCGGGAAGACTGT	*hrcT* gene fragment, 104bp
hrcU-F	CAGGCACGCAGCCAGGAA	
hrcU-R	GCGATGCAACGGCGATAA	*hrcU* gene fragment, 86bp
hrpE-F	CGTTGTCGCCCGCCTT	
hrpE-R	GGTTCGTTGCTCGGCG	*hrpE* gene fragment, 134bp
hrpF-F	AGTCCGGCGTGCTCATCG	
hrpF-R	AGTGCCCACCGCAGTTGA	*hrpF* gene fragment, 102 bp
gyrB-F	GGCGAGCACAATGGCATT	
gyrB-R	CCATCCTTCTGCGGGATGT	*gyrB* gene fragment, 101bp
